# Training Spiking Neural Networks for Reinforcement Learning Tasks With Temporal Coding Method

**DOI:** 10.3389/fnins.2022.877701

**Published:** 2022-08-17

**Authors:** Guanlin Wu, Dongchen Liang, Shaotong Luan, Ji Wang

**Affiliations:** ^1^Academy of Military Science, Beijing, China; ^2^Nanhu Laboratory, Jiaxing, China; ^3^College of Systems Engineering, National University of Defense Technology, Changsha, China

**Keywords:** spiking neural networks, reinforcement learning, temporal coding, fully differentiable, asynchronous processing

## Abstract

Recent years witness an increasing demand for using spiking neural networks (SNNs) to implement artificial intelligent systems. There is a demand of combining SNNs with reinforcement learning architectures to find an effective training method. Recently, temporal coding method has been proposed to train spiking neural networks while preserving the asynchronous nature of spiking neurons to preserve the asynchronous nature of SNNs. We propose a training method that enables temporal coding method in RL tasks. To tackle the problem of high sparsity of spikes, we introduce a self-incremental variable to push each spiking neuron to fire, which makes SNNs fully differentiable. In addition, an encoding method is proposed to solve the problem of information loss of temporal-coded inputs. The experimental results show that the SNNs trained by our proposed method can achieve comparable performance of the state-of-the-art artificial neural networks in benchmark tasks of reinforcement learning.

## 1. Introduction

Neuromorphic engineering aims to emulate the dynamics of biological neurons and synapses with silicon circuits and run spiking neural networks (SNNs) to achieve cognitive behaviors (Mead, 1990). SNNs enjoy the advantages of the unique computing architecture of the brain, such as low-power consumption, massive parallelism, and low-latency processing.

Among the recently proposed training methods of SNNs (Zhang and Li, 2019, 2020; Comsa et al., 2020; Kim et al., 2020; Li and Pehlevan, 2020), the temporal coding (TC) method (Mostafa, 2017; Comsa et al., 2020) emerges as a promising one by achieving state-of-the-art performance in many tasks while preserving the asynchronous processing nature of biological spiking neurons. The TC method bridges the gap between artificial neural networks (ANNs) and SNNs. It encodes neural dynamics as a relation of pre-synaptic spike times and the spike time of a neuron. Back-propagation techniques developed for ANNs can thus be used in SNNs while preserving the network's capability of fast response to sensory stimuli. Nonetheless, all the existing TC methods are designed for classification tasks, e.g., Boolean logic tasks and image recognition tasks (Mostafa, 2017; Comsa et al., 2020), not fitting well with reinforcement learning tasks which require the input and output of SNNs are continuous values.

While there is an increasing demand for applying SNNs to reinforcement learning (RL) tasks (Tang et al., 2020a,b), there is no reported trial of using temporal coding methods to train neural networks in RL tasks. Many advances have been made on the training of SNNs for reinforcement learning tasks. Most of the current works use the rate coding method (Patel et al., 2019; Tang et al., 2020a,b; Tan et al., 2021) to train neurons, and regard SNNs as a synchronous system where neurons are activated layer by layer like ANNs (Rosenfeld et al., 2019). Disappointingly, the SNNs trained by these methods cannot be directly deployed on the latest asynchronous neuromorphic processors that faithfully emulate the spike coding and asynchronous nature of the biological neural systems, such as Dynapse, DynapCNN, or Loihi. There is a necessary conversion step between the ANNs or rate-based networks to SNNs, and then they can be deployed to those chips. In our methods, training with temporal coding can avoid this conversion step.

Compared with temporal coding SNNs, the cost of currently the most widely used rate coding SNNs mainly lies in response delay and accuracy. The spiking neural network based on temporal coding can cleverly use the activation time of the input layer to represent information, which means an inference can be completed in one activation cycle. Rate coding SNNs need to estimate information based on the activation frequency over a period of time, which takes more time and loses accuracy. In addition, the transcoding process also loses accuracy, which is not the case with temporal coding SNNs.

But to train SNNs for reinforcement learning tasks with the TC method, there are two critical challenges when the input and output of SNNs are continuous values. Firstly, the derivative of the current temporal-coded SNNs does not exist everywhere in the network during training, which deteriorates the performance of back-propagation training for RL tasks. Without ensuring the existence of the derivative of SNNs everywhere, the existing TC methods cannot converge for RL tasks. Secondly, due to the intrinsic computing paradigm of SNNs, if an input signal is encoded as a relatively large value, especially when it arrives after the first output neuron spikes, it cannot effectively participate in the training and inference of SNNs. A sophisticated signal encoding method is required to transfer input signals to spike times in a restricted range to ensure the effective usage of all the input signals.

Inspired by the excellent performance of the TC method, we attempt to apply it to reinforcement learning tasks in this work. We propose a Continuous-Valued Temporal Coding (CVTC) method to tackle the above-motioned challenges. To the best of our knowledge, this is the first work to train SNNs with temporal coding methods for RL tasks. The main contributions are as follows:

- We design a fully differentiable temporal-coded SNN architecture (see Section 3). By introducing a self-incremental factor to each spiking neuron, the proposed SNN architecture ensures that each neuron is differentiable almost anytime and everywhere during training.
- We propose a signal encoding method for continuous input signals (see Section 4). Based on a mixture of spatial and temporal coding techniques, the novel encoding method can transform input signals to spike times and solve the problem of losing information of later arrived spikes.
- Experimental results show the effectiveness of the proposed CVTC method for RL tasks (see Section 5). The SNN trained by the CVTC method achieves a comparable performance of the state-of-the-art ANN in the DDQN framework with the same number of network parameters.

## 2. Background

Most of the studies on temporal coding methods focus on how to transform spiking neurons' input spike times to their output spike times and calculate derivatives (Neftci et al., 2019). There are three typical methods (Bohte et al., 2002; Mostafa, 2017; Comsa et al., 2020). SpikeProp (Bohte et al., 2002) is believed the first temporal coding method for training SNNs, where sub-connections are used for each pair of connected neurons to transform input spike times to output spike times. Instead, Mostafa (2017) relies on simple neural and synaptic dynamics, resulting in an analytical relation between input and output spike times. The SNNs thus can be trained with commonly used GPU-accelerated ANN training packages. Recently, Comsa et al. (2020) used an Alpha Synaptic Function to construct spiking neurons inspired by biological evidence.

Among these three methods, Mostafa (2017) is the most typical one of temporal coding. Our method is designed mainly based on the study from Mostafa (2017). Here we briefly introduce the network model proposed in Mostafa (2017). The author used non-leaky I&F neurons with exponentially decaying synaptic current kernels. The dynamics of neuron's membrane potential are described as:


(1) $$\begin{array}{l} \frac{dV_{mem}\left(t\right)}{dt}=\underset{i}{\sum }w_{i}\underset{r}{\sum }\kappa \left(t-t_{i}^{r}\right), \end{array}$$

where $\kappa \left(x\right)=\Theta \left(x\right)exp\left(-\frac{x}{\tau_{syn}}\right)$, where $\Theta (x)=\{\begin{array}{ll} 1 & \text{if }x\geq 0\\ 0 & \text{otherwise} \end{array}$. Assume the neuron spikes in response at time *t*_*out*_. By integrating Equation (1), the membrane potential for *t* < *tout* is given by:


(2) $$\begin{array}{l} V_{mem}\left(t\right)=\overset{N}{\underset{i=1}{\sum }}\Theta \left(t-t_{i}\right)w_{i}\left(1-exp\left[-\left(t-t_{i}\right)\right]\right). \end{array}$$

The neuron spikes when its membrane potential crosses a firing threshold which is set as 1. Then the spike time *t*_*out*_ is implied as:


(3) $$\begin{array}{l} 1=\underset{i\in C}{\sum }w_{i}\left(1-exp\left[-\left(t_{out}-t_{i}\right)\right]\right), \end{array}$$

where *C* = {*i*:*t*_*i*_ < *t*_*out*_} is the subset of input spikes which actually affect the output neuron. Eventually, the exponential form of *t*_*out*_ can be denoted as:


(4) $$\begin{array}{l} exp\left(t_{out}\right)=\frac{\sum_{i\in C}w_{i}exp\left(t_{i}\right)}{\sum_{i\in C}w_{i}-1}. \end{array}$$

If *exp*(*t*_*out*_) is denoted as *z*_*out*_, the input and output relation of a spiking neuron can thus be transformed to the same form as a typical artificial neuron. In this way, the back-propagation technique can be used for training SNNs.

In the temporal coding method, to ensure the back-propagation work normally and effectively and the output neurons emit spikes, the following conditions need to be guaranteed:


(5) $$\begin{array}{l} \underset{i\in C}{\sum }w_{i}>1, \end{array}$$

(6) $$\begin{array}{l} exp\left(t_{out}\right)>1. \end{array}$$

Otherwise, the *exp*(*t*_*out*_) would be set to INF. We notice that due to the sparsity of spikes in SNNs, most of the neuron outputs would be set to INF. In the next section, we present the proposed training method based on the equations above.

## 3. Fully Differentiable Temporal-Coding Training Method

The current TC method uses back-propagation technique to train SNNs for classification tasks. However, for general RL frameworks, such as Deep Q Network (DQN) (Fan et al., 2020) and Actor-Critic (AC) (Degris et al., 2012), it requires at least one neural network to regress the *Q*-value of states. Based on our preliminary experiments, we find that during training an SNN for regression tasks, there is always a case that some neurons emit spikes with relatively short spike time (predicted value), and many of the other neurons emit spikes with INF (assigned value). The network parameters are updated with the back-propagation algorithm based on the output neurons emitting predicted spike times. The neurons with INF times would be ignored. Hence the parameters of SNN may not be updated normally when using the TC method proposed in Mostafa (2017).

Taking *Q*-value prediction as an example, Table 1 describes the results of the back-propagation algorithm in four different situations about the network output *Q*(*s*) and the target *Q*(*s*′)+*r*. When *Q*(*s*) and *Q*(*s*′)+*r* are normal, the backpropagation runs normally. When *Q*(*s*) is INF, the derivative of the feed-forward network is 0, and the loss will not be fed back. When *Q*(*s*) is normal but *Q*(*s*′)+*r* is INF, the feed-forward network's derivative exists, and INF will be fed back to the network and unable to train normally. In the case of **stop**, the network cannot be updated, resulting in a fixed output value. In the case of **error**, there will be unforeseen circumstances. Neither of them is expected to appear in the *Q*-value prediction of RL tasks.

**Table 1:** Back-propagation cases in RL tasks.

***Q*(*s*)**	***Q*(*s*′)+*r***	**Derivative**	**Back-propagation**
Legal	Legal	Exist	Normal
INF	Legal	Equal to 0	Stop
INF	INF	Equal to 0	Stop
Legal	INF	Exist	Error

To tackle the above problem of derivative discontinuity, we propose a fully differentiable temporal-coding training method in the following part. Section 3.1 introduces a self-incremental variable to make the TC method fully differentiable. Section 3.2 further discusses the impact of the self-incremental variable during the inference phase of trained SNNs.

### 3.1. Training SNNs With a Self-incremental Variable

To solve the problem above, here we modify the spiking neuron model and introduce a self-incremental variable β*exp*(*t*) for each of the spiking neurons. It ensures that every neuron will be activated in a limited time, and its derivative is always continuous for all the possible inputs. Without adding this term, a neuron's output spike time could be set to INF according to Mostafa (2017) and its derivative will be 0. The SNN could not be successfully updated during training through back propagation. Thus, the dynamics of a spiking neuron's membrane potential can be described as:


(7) $$\begin{array}{l} \frac{dV_{mem}\left(t\right)}{dt}=\underset{i}{\sum }w_{i}\underset{r}{\sum }\kappa \left(t-t_{i}^{r}\right)+\beta exp\left(t\right). \end{array}$$

By integrating Equation (7), the spike time *t*_*out*_ can be implied as:


(8) $$\begin{array}{l} 1=\underset{i\in C}{\sum }w_{i}\left[1-exp\left(-t_{out}+t_{i}\right)\right]+\beta exp\left(t_{out}\right)-\beta , \end{array}$$

where β is a hyperparameter. Hence, the exponential form of *t*_*out*_ can be calculated with:


(9) $$\begin{array}{l} exp\left(t_{out}\right)=\sqrt{\frac{\sum_{i\in C}w_{i}exp\left(t_{i}\right)}{\beta }+\frac{{\left(1+\beta -\sum_{i\in C}w_{i}\right)}^{2}}{4\beta^{2}}}\\ \;+\frac{1+\beta -\sum_{i\in C}w_{i}}{2\beta }, \end{array}$$

where the following requirements has to be satisfied:


(10) $$\begin{array}{l} {\left(\underset{i\in C}{\sum }w_{i}-1-\beta \right)}^{2}>-4\beta \underset{i\in C}{\sum }w_{i}exp\left(t_{i}\right), \end{array}$$

(11) $$\begin{array}{l} exp\left(t_{out}\right)>1. \end{array}$$

Otherwise, the *exp*(*t*_*out*_) would be set to INF. Our proposed temporal coding method ensures that the derivative for each neuron is always continuous as long as Equation (10) is satisfied.

For the convenience of comparison, we illustrate our algorithm using the same style as Mostafa (2017), where a transformation of variables is made: *exp*(*t*_*k*_) → *z*[*k*]. Algorithm 1 is the pseudocode of the forward pass, where *get*_*causal*_*set* is a function that gets indices of input spikes influencing the spike time of the first output of a neuron, as shown in Algorithm 2.

**Algorithm 1:** Pseudocode of the forward pass in a feed-forward network with L layers.

**Input**: *z^0^: Vector of input spike times encoded with Algorithm 1*
**Input**: *N*^1^, ..., *N^L^: Number of neurons in the L layers*
**Input**: *W*^1^, ..., *W^L^: Set of weight matrices. W^l^[i, j] is the weight from neuron j in layer l* − 1 *to neuron i in layer l*
**Output**: *z^L^: Vector of first spike times of neurons in the output layer*
1: **for** *r* = 1 to *L* **do**
2: **for** *i* = 1 to *N^r^* **do**
3: $C_{i}^{r}\leftarrow get\text{\_}causal\text{\_}set\left(t^{r-1},W^{r}\left[i,:\right]\right)$
4: **if** $C_{i}^{r}\neq \phi$ **then**
5: $z^{r}\left[i\right]\leftarrow \sqrt{\frac{\overset{i}{\underset{k=1}{\sum }}w_{k}z_{k}^{r-1}}{\beta }+\frac{{\left(1+\beta -\overset{i}{\underset{k=1}{\sum }}w_{k}\right)}^{2}}{4\beta^{2}}}+\frac{1+\beta -\overset{i}{\underset{k=1}{\sum }}w_{k}}{2\beta }$
6: **else**
7: *z^r^*[*i*] ← ∞
8: **end if**
9: **end for**
10: **end for**

**Algorithm 2:** Pseudocode of the get_causal_set function.

**Input**: *z*: *Vector of input spike times of length N*
**Input**: *w*: *Weight vector of the input spikes*
**Output**: *C*: *Causal index set*
1: *sort_indices ← argsort(z) //Ascending order argsort*
2: *z^sorted^ ← z[sort_indices] //sorted input vectors*
3: *w^sorted^ ← w[sort_indices] //weight vector rearranged to match sorted input vector*
4: **for** *i* = 1 to *N* **do**
5: **if** *i* = = *N* **then**
6: *next_input_spike* ← ∞
7: **else**
8: *next_input_spike* ← *z^sorted^*[*i* + 1]
9: **end if**
10: **if** ${\left(\overset{i}{\underset{k=1}{\sum }}w^{sorted}\left[k\right]-1-\beta \right)}^{2}>-4\beta \overset{i}{\underset{k=1}{\sum }}w^{sorted}\left[k\right]z^{sorted}\left[k\right]\wedge 1<\sqrt{\frac{\overset{i}{\underset{k=1}{\sum }}w^{sorted}{\left[k\right]}^{sorted}\left[k\right]}{\beta }+\frac{{\left(1+\beta -\overset{i}{\underset{k=1}{\sum }}w^{sorted}\left[k\right]\right)}^{2}}{4\beta^{2}}}+\frac{1+\beta -\overset{i}{\underset{k=1}{\sum }}w^{sorted}\left[k\right]}{2\beta }<next\text{\_}input\text{\_}spike$ **then**
11: **return** *sort_indices*[1], ..., *sort_indices*[*i*]
12: **end if**
13: **end for**
14: **return** ϕ

**Figure 1:**
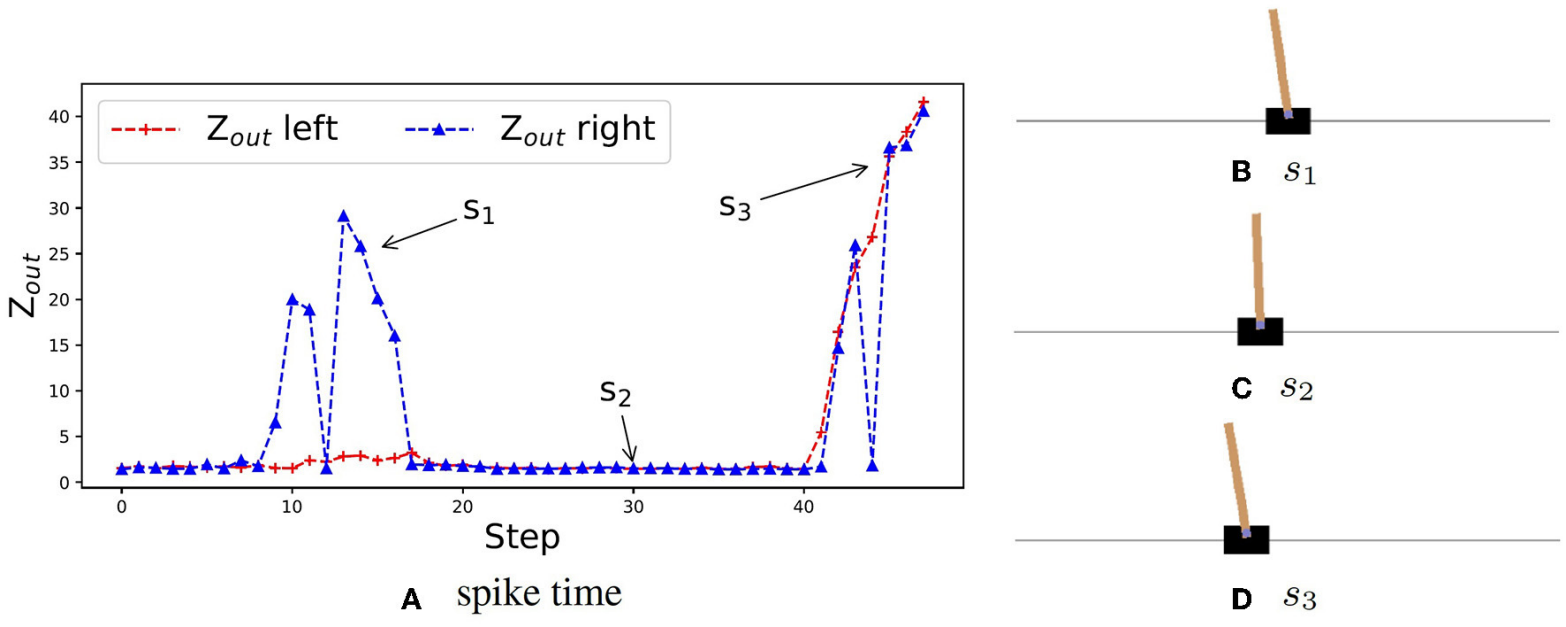
Spike time variation of the output layer of CVTC. **(A)** An example of spike time in an episode shows the neurons' spike time for moving left and right of each step. **(B)** States of the car at step *s*_1_. The car is at the center of the field, and the pendulum is turning left. The spike time for moving right is high. **(C)** States of the car at step *s*_2_. The car is at the center of the field, and the pendulum is upright. The spike times are both low. **(D)** States of the car at step *s*_3_. The car is on the left of the field, and the pendulum is turning left. The spike times are both high.

A reinforcement learning problem can converge only if the *Q*-value has maximum. It allows the z-domain (from 1 to ∞) to contain the range of *Q*-values. Then we can use the spike time in the z-domain to represent the *Q*-value of RL. To better illustrate that the proposed method can generate a practical predictive value, we visualize the spike time variation of the output layer in the CartPole task. As shown in Figure 1, around step *s*_1_, the pendulum tilts left, the neuron for moving left keeps spiking faster than that of moving right. The network can keep selecting the left-moving action until the upright state is restored. Around step *s*_2_, the pendulum is upright, the neuron for moving left and right both spike quickly, which means the expectation is always high, and thus this state is close to the ideal one. At step *s*_3_, the pendulum has been shifted to the left of the field and tilts to the left. Since the network has not been well trained for this situation yet, the two output neurons' spike order flips between steps. The network cannot continue to select the expected action, left moving, to restore the upright state. It shows that our training method is effective and in line with expectations.

### 3.2. Inference Without the Self-Incremental Variable

In the above section, we introduce a self-incremental variable. In this section, we discuss the impact of this variable when inferencing the trained SNN on real chips. Although the self-incremental variable is usually easy to implement with mixed-signal analog/digital circuits, we further explore how to deploy the trained network on neuromorphic hardware without dedicated modification. Therefore, in the inference stage of a trained SNN, the implementation of this variable is removed. The method given in this section is to directly use Equation (4) to calculate the activation time during inference. Since β in Equation (9) is small enough, when the DQN algorithm converges, the difference between Equations (4) and (9) approaches zero.

**Theorem 1**. *In Q network of CVTC method, p_j_ is the jth neuron's spike time of output layer using Equation (9), and q_j_ is the jth neuron's spike time of output layer using Equation (4). For any ϵ > 0, there is a small enough β for all j such that*:


(12) $$\begin{array}{l} |exp\left(q_{j}\right)-exp\left(p_{j}\right)|<\epsilon . \end{array}$$

During the training phase of SNNs, we set β as a small enough value and use Equation (9) to calculate the spike time. During the inference phase on real chips, we ignore the self-incremental variable and directly use Equation (4) to implement the circuit. According to the experimental results in Section 5.1, when β is set as a value smaller than 1*e* − 2, the performance degradation caused by ignoring the self-incremental variable is negligible.

## 4. Encode Input Signals for Temporally Coded Spiking Neural Networks

**Figure 2:**
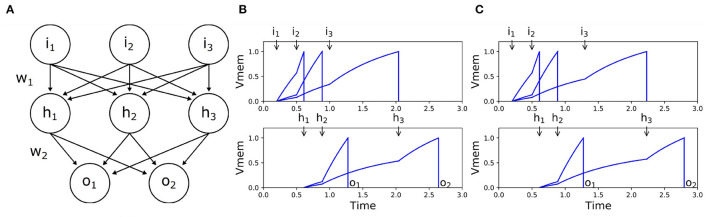
Example of the unbalanced-input problem in SNNs. **(A)** Network structure. **(B)** Input-output spike times when *i*_3_ = 1.0. **(C)** Input-output spike times when *i*_3_ = 1.3. Since we take the first activated output neuron as the network's output, it cannot distinguish the two different input patterns in this example. When we delayed *i*_3_ from 1.0 **(B)** to 1.3 **(C)**, it only affected *o*_2_.

Temporal-coded input information's contribution is inherently biased in asynchronous SNNs. In such networks, the input spikes that arrive earlier affect the processing of the subsequent spikes. Thus, the earlier spikes have a higher impact on the SNNs's output, which is undesirable. In reinforcement learning tasks, the input signal represents the observation of the environment, such as how far the agent is from the center, how large the angle is, how large the speed is, etc. When we transfer the value to a spike timing, ideally, the timing should not have any predefined impact factor because we are not sure if the observed value should be larger or lower. This should be the task for the reinforcement learning algorithm to discover. Figure 2 shows one case of this phenomenon where the last input spikes have no impact on the network's output. It is unfeasible to directly feed the input signals as spike times into asynchronous SNNs.

To solve this problem of the inherently biased contribution of input signals, we propose an encoding method based on a mixture of spatial and temporal coding techniques, which can solve the problem largely while keeping the input information intact during the coding procedure. In Section 4.1, we present our encoding method for input signals of SNNs. In Section 4.2, we prove that the encoded input signal can be easily recovered, and there is no information loss during encoding.

### 4.1. Encode With Neuron Populations and Normal Distribution

We discrete the range [*a, b*] of value *x* into *K* points *k* ∈ [0, *K* − 1]. For an input channel *i* ∈ *I*, if a spike is generated at time *x*_*i*_, we have


(13) $$\begin{array}{l} s_{i,k}=\{\begin{array}{ll} T, & Floor(\frac{x_{i}-a_{i}}{b_{i}-a_{i}}*K)=k\\ 0, & otherwise \end{array}, \end{array}$$

where *s*_*i,k*_ is the value of the k-th point of i-th input channel. *T* is a default spike time. All of the *I* × *K* all-or-none input channels are fed into the input layer of SNNs with spike time *T* or 0.

Thus, continuous temporal signals can be mapped into discrete spatial signals, and they can be treated equally by the network as different parts of an input image. However, this coding method is achieved at the cost of losing precision, making it unable to distinguish subtle differences between input signals and only representing 2^*K*^ different input values.

Then we extend the coding method presented in this section to take advantage of a normal distribution of μ = *x* to determine the timing value of each point. In this way, the input signal can be encoded as spikes with continuous times, which can be easily decoded to the original information it carries. Now the input signal can be encoded as:


(14) $$\begin{array}{l} s_{i,k}=\left[\frac{1}{\sqrt{2\pi }\sigma }-Norm_{\frac{x_{i}-a_{i}}{b_{i}-a_{i}}*K,\sigma^{2}}\left(k\right)\right]*T, \end{array}$$

where *Norm* is the probability density function of the normal distribution. σ is the pulse width, can always be set as 1.

In this way, the original input signal is encoded as continuous spike time in the range of [*a, b*]. *K* is the input width after conversion. The larger the width, the more information the input contains, so it should be as large as possible within the range that the network performance can bear. We set *K* to 20 in the following experiments of this paper.

It worth noting that since the final original output activation time cannot be recovered from more than two output signals when the output is mixed with noise, the premise that the signal can be recovered in Section 4.1 must be lossless, and the method in Section 4.1 cannot be directly applied to the output. In addition, it is also difficult to implement the *Q*-value function of reinforcement learning tasks with discretization method. So we can only directly map the activation time of the output layer to the *Q*-value. But relying on the method in Section 3.1 to enhance the continuity of backpropagation, our method has been able to enable the agent to overcome the imbalance of the output and finally complete the training task as shown in the experimental results below.

The pseudocode of our encoding method presented is illustrated in Algorithm 3.

**Algorithm 3:** Pseudocode for encoding the input signals.

**Input**: (*x*_1_, .., *x_L_*): *Input signals*
**Output**: (*y*_1_, .., y_L*K_): *Encoded spike times*
1: *a* ← *min*(*x*_1_, .., *x_L_*)
2: *b* ← *max*(*x*_1_, .., *x_L_*)
3: **for** *i* = 1 → *L* **do**
4: **for** *k* = 1 → *K* **do**
5: $S_{i,k}\leftarrow \left(1-\frac{1}{\sqrt{2\pi }\sigma }exp\left[-\frac{{\left(k-\frac{x_{i}-a}{b-a}*K\right)}^{2}}{2\sigma^{2}}\right]\right)$
6: Y_i*K+k_ ← S_i,k_
7: **end for**
8: **end for**

### 4.2. Recoverable Encoded Input Signal

Here we show that the input encoding method in Section 4.1 is non-destructive and can be recovered to the original input. The probability density function of the normal distribution in Equation (14) is defined as:


(15) $$\begin{array}{l} Norm_{\mu ,\sigma^{2}}\left(k\right)=\frac{1}{\sqrt{2\pi }\sigma }exp\left[-\frac{{\left(k-\mu \right)}^{2}}{2\sigma^{2}}\right]. \end{array}$$

Substitute the term of normal distribution in Equation (14) with Equation (15), the encoded input signal becomes:


(16) $$\begin{array}{l} s_{i,k}=\left[1-\frac{1}{\sqrt{2\pi }\sigma }exp\left(-\frac{{\left(k-\frac{x_{i}-a_{i}}{b_{i}-a_{i}}*K\right)}^{2}}{2\sigma^{2}}\right)\right]*T, \end{array}$$

Hence, the origin input signal are given by:


(17) $$\begin{array}{l} x_{i}=\left(k\pm \sqrt{2\sigma^{2}ln\left[\sqrt{2\pi }\sigma \left(1-\frac{s_{i,k}}{T}\right)\right]}\right)/K*\left(b_{i}-a_{i}\right)+a_{i}. \end{array}$$

Based on Equation (17), *x*_*i*_ can be easily recovered from the encoded input.

### 4.3. Comparison of Different Self-incremental Variables

The membrane potential of a spiking neuron when that is about to fire is described as:


(18) $$\begin{array}{l} \theta =\underset{i\in C}{\sum }w_{i}\left(1-exp\left[-\left(t_{out}-t_{i}\right)\right]\right)+\int_{0}^{t_{out}}f\left(x\right)\text{d}x, \end{array}$$

where *f*(*x*) is the self-incremental variable that we introduced. With this term, the membrane potential can continue to increase over time. It makes sure that every neuron can reach the firing threshold eventually so that no neuron's output spike would be denoted as INF. When the input spikes are not given, to ensure the neuron can spike eventually, it requires:


(19) $$\begin{array}{l} \int_{0}^{\infty }f\left(x\right)\text{d}x>=\theta \;\left[f\left(x\right)>0\right]. \end{array}$$

We can transform Equation (18) to:


(20) $$\begin{array}{l} \alpha \frac{1}{exp\left(t_{out}\right)}+\gamma +\int_{0}^{t_{out}}f\left(x\right)\text{d}x=0, \end{array}$$

where α and γ denote two constants. Then we have:


(21) $$\begin{array}{l} \int_{0}^{t_{out}}f\left(x\right)\text{d}x=\eta exp{\left(t_{out}\right)}^{k}+C\;\left(k\in \mathbb{Z}+\right), \end{array}$$

where *f*(*x*) has multiple candidates. We choose to set *f*(*x*) as β*exp*(*x*). Because only in this case, Equation (21) is not a transcendental equation, and it can be solved using common back-propagation algorithms. In the other cases, Equation (21) would be a transcendental equation, to deal with it, we have to use one of the following methods:

- Use iterative algorithms such as Newton's method to get a numerical solution. However, this would result in a great reduce of the efficiency of the solution.
- Use low-order Taylor expansion approximation. However, this would result in an accuracy-decreasing problem.

Therefore, we choose to use β*exp*(*x*) as the self-incremental variable to smooth the training process.

### 4.4. Architectures

In this paper, we use full-connected structure as the temporal-layers. For MNIST task, we use the same network structure with one hidden layer. Hidden layers of both the CVTC and Temporal Coding (TC) network have 800 neurons. For CartPole task, we also use one hidden layer of 800 neurons for all SNN networks. But the input sizes of DDQN-SNN-CVTC and DDQN-SNN-TC-encoded are 80 instead of 4. For MountainCar task, all networks have two hidden layers of 12 and 48 layers, including SNN and ANN methods. The input size of DDQN-SNN-CVTC are expanded to 40 by input encoding.

### 4.5. Hyperparameters

In Table 2, the Hyperparameters for our experiments in this paper are shown.

**Table 2:** Hyperparameters for algorithms in experiments.

**Hyperparameter**	**MNIST**	**CartPole**	**MountainCar**
Optimization algorithm	SGD (Amari, 1993)	Adam (Kingma and Ba, 2014)	Adam
Learning rate	0.01–0.0001	0.001251	0.001
Training batch size	10	32	32
Target network update frequency		100 step	1 episode
Replay memory capacity		1,000	200,000
Training batch size	23.37	−200	−106.4
γ		0.99	0.99
ϵ		1–0.1	1–0.00001
*K*		20	15
*T*		20	15
σ		1.4	1.2
β	0.1, 0.01, 0.001	0.001	0.001

### 4.6. Inference With I&F Neurons

Here we show that the added incremental term can be removed after training. So the trained network can be run in typical SNNs constructed with I&F neurons.

**Theorem 1**. *In Q network of CVTC method*, *p*_*j*_ *is the* *jth* *neuron's spike time of output layer using Equation (9), and* *q*_*j*_ *is the* *jth* *neuron's spike time of output layer using Equation (4). For any* ϵ > 0, *there is a small enough* β *for all* *j* *such that:*


(22) $$\begin{array}{l} |exp\left(q_{j}\right)-exp\left(p_{j}\right)|<\epsilon . \end{array}$$

When the DQN algorithm converges, the predicted value of the output layer is upper bound. Let *L* donate the layer number of Q network, *pred*_*max*_ donate the max activation time of the output layer in the Q network. As we discussed at the beginning of Section 4, in each layer of the network, only those inputs that are less than the maximum spike time of the output layer could affect the output layer. Let *p*_*l,j*_ donate the *jth* neuron's spike time of layer *l* + 1 and *p*_*l,j*_ < *pred*_*max*_.

It can be conducted that all output times are positive as follows:


(23) $$\begin{array}{l} exp\left(p_{l,j}\right)>1. \end{array}$$

So we always have:


(24) $$\begin{array}{l} exp\left(p_{l,j}\right)<\frac{\sum_{i\in C}w_{l,i}exp\left(p_{l-1,j}\right)}{\sum_{i\in C}w_{l,i}-1}=exp\left(q_{l,j}\right), \end{array}$$

where *C* = {*i*:*p*_*l* − 1, *j*_ < *p*_*l,j*_}, *w*_*l,i,j*_ donate the weight between neuron *i* of layer *l* and neuron *j* of layer *l* + 1. Thus we have:


(25) $$\begin{array}{l} exp\left(q_{l,j}\right)-exp\left(p_{l,j}\right)>0. \end{array}$$

For the first layer of Q network, inputs for *p*_0,*j*_ and *q*_0,*j*_ are same. it can be obtained by Equation (8):


(26) $$\begin{array}{l} \begin{array}{c} 1=\underset{i\in C}{\sum }w_{0,i,j}\left[1-exp\left(-p_{0,j}+t_{i}\right)\right]+\beta exp\left(p_{0,j}\right)-\beta \\ <\underset{i\in C}{\sum }w_{0,i,j}\left[1-exp\left(-p_{0,j}+t_{i}\right)\right]+\beta exp\left(q_{0,j}\right)-\beta , \end{array} \end{array}$$

where *t*_*i*_ donate the *ith* input of Q network.

Simplify the equation:


(27) $$\begin{array}{l} exp\left(p_{0,j}\right)\left[1+\beta -\beta exp\left[q_{0,j}\right]-\underset{i\in C}{\sum }w_{0,i,j}\right]<\underset{i\in C}{\sum }w_{0,i,j}exp\left(t_{i}\right), \end{array}$$

(28) $$\begin{array}{l} exp\left(p_{0,j}\right)\left[\beta exp\left[q_{0,j}\right]+\underset{i\in C}{\sum }w_{0,i,j}-1-\beta \right]>\underset{i\in C}{\sum }w_{0,i,j}exp\left(t_{i}\right). \end{array}$$

The lower bound of *exp*(*p*_0,*j*_) can be obtained:


(29) $$\begin{array}{l} exp\left(p_{0,j}\right)>\frac{\sum_{i\in C}w_{0,i,j}exp\left(t_{i}\right)}{\sum_{i\in C}w_{0,i,j}+\beta exp\left(q_{0,j}\right)-1-\beta }. \end{array}$$

Subtract *exp*(*q*_0,*j*_) on both sides:


(30) $$\begin{array}{l} \begin{array}{c} exp\left(q_{0,j}\right)-exp\left(p_{0,j}\right)<\frac{\sum_{i\in C}w_{0,i,j}exp\left(t_{i}\right)}{\sum_{i\in C}w_{0,i,j}-1}\\ -\frac{\sum_{i\in C}w_{0,i,j}exp\left(t_{i}\right)}{\sum_{i\in C}w_{0,i,j}+\beta exp\left(q_{0,j}\right)-1-\beta }\\ =\frac{\sum_{i\in C}w_{0,i,j}exp\left(t_{i}\right)\left[\left(\sum_{i\in C}w_{0,i,j}+\beta exp\left(q_{0,j}\right)-1-\beta \right)-\left[\sum_{i\in C}w_{0,i,j}-1\right]\right]}{\left(\sum_{i\in C}w_{0,i,j}-1\right)\left[\sum_{i\in C}w_{0,i,j}+\beta exp\left(q_{0,j}\right)-1-\beta \right]}\\ =\frac{\sum_{i\in C}w_{0,i,j}exp\left(t_{i}\right)\left[\beta exp\left(q_{0,j}\right)-\beta \right]}{\left(\sum_{i\in C}w_{0,i,j}-1\right)\left[\sum_{i\in C}w_{0,i,j}+\beta exp\left(q_{0,j}\right)-1-\beta \right]}\\ =\frac{AB}{W\left(W+B\right)}\\ <\frac{AB}{W^{2}}, \end{array} \end{array}$$

where $A=\underset{i\in C}{\sum }w_{0,i,j}exp\left(t_{i}\right)$, $W=\underset{i\in C}{\sum }w_{0,i,j}-1$ and *B* = β*exp*(*q*_0,*j*_) − β, A, B, and W are all positive. Let:


(31) $$\begin{array}{l} exp\left(q_{0,j}\right)-exp\left(p_{0,j}\right)<\epsilon , \end{array}$$

β should satisfy:


(32) $$\begin{array}{l} \beta <\frac{\epsilon W^{2}}{A\left[exp\left(q_{0,j}\right)-1\right]}. \end{array}$$

So when we choose $\beta =\frac{\epsilon W^{2}}{2^{L}A\left[exp\left(pred_{max}\right)-1\right]}$, there is:


(33) $$\begin{array}{l} exp\left(q_{0,j}\right)-exp\left(p_{0,j}\right)<\epsilon \frac{exp\left(q_{0,j}\right)-1}{2^{L}exp\left(pred_{max}\right)-1}. \end{array}$$

Thus we proved the limit of loss for one layer. Then we generalize it for all layers. For layer *l* > 0, let $\beta_{l,j}=\frac{\epsilon W_{l,j}^{2}}{2^{L-l}A_{l,j}\left[exp\left(pred_{max}\right)-1\right]}$. If $exp\left(q_{l-1,j}\right)-exp\left(p_{l-1,j}\right)<\epsilon \frac{exp\left(q_{0,j}\right)-1}{2^{L-l}exp\left(pred_{max}\right)-1}$ holds, then we rewrite Equation (30) as:


(34) $$\begin{array}{l} \begin{array}{c} exp\left(q_{l,j}\right)-exp\left(p_{l,j}\right)<\frac{\sum_{i\in C}w_{l,i}exp\left(q_{l-1,j}\right)}{\sum_{i\in C}w_{l,i}-1}\\ -\frac{\sum_{i\in C}w_{l,i}exp\left(p_{l-1,j}\right)}{\sum_{i\in C}w_{l,i}+\beta exp\left(q_{l,j}\right)-1-\beta }\\ <\frac{\sum_{i\in C}w_{l,i}\left(exp\left(p_{l-1,j}\right)+\epsilon \frac{exp\left(q_{l-1,j}\right)-1}{2^{L-l}exp\left(pred_{max}\right)-1}\right)}{\sum_{i\in C}w_{l,i}-1}\\ -\frac{\sum_{i\in C}w_{l,i}exp\left(p_{l-1,j}\right)}{\sum_{i\in C}w_{l,i}+\beta exp\left(q_{l,j}\right)-1-\beta }\\ =\frac{\sum_{i\in C}w_{l,i}exp\left(p_{l-1,j}\right)\left[\beta exp\left(q_{l,j}\right)-\beta \right]}{\left(\sum_{i\in C}w_{l,i}-1\right)\left[\sum_{i\in C}w_{l,i}+\beta exp\left(q_{l,j}\right)-1-\beta \right]}\\ +\frac{\epsilon }{\sum_{i\in C}w_{l,i}-1}\frac{\sum_{i\in C}w_{l,i}exp\left(q_{l-1,j}\right)-1}{2^{L-l}exp\left(pred_{max}\right)-1}, \end{array} \end{array}$$

(35) $$\begin{array}{l} \begin{array}{c} exp\left(q_{l,j}\right)-exp\left(p_{l,j}\right)<\epsilon \frac{q_{l,j}}{2^{L-l}exp\left(pred_{max}\right)}\\ +\frac{\epsilon }{\sum_{i\in C}w_{l,i}-1}\frac{\sum_{i\in C}w_{l,i}exp\left(q_{l-1,j}\right)-1}{2^{L-l}exp\left(pred_{max}\right)-1}\\ <\epsilon \frac{q_{l,j}}{2^{L-l}exp\left(pred_{max}\right)}+\frac{\epsilon }{\sum_{i\in C}w_{l,i}-1}\\ \frac{\sum_{i\in C}w_{l,i}exp\left(q_{l-1,j}\right)}{2^{L-l}exp\left(pred_{max}\right)}\\ =\epsilon \frac{q_{l,j}}{2^{L-l}exp\left(pred_{max}\right)}+\frac{\epsilon }{2^{L-l}exp\left(pred_{max}\right)}\\ \frac{\sum_{i\in C}w_{l,i}exp\left(q_{l-1,j}\right)}{\sum_{i\in C}w_{l,i}-1}\\ =\epsilon \frac{q_{l,j}}{2^{L-l}exp\left(pred_{max}\right)}+\frac{\epsilon }{2^{L-l}exp\left(pred_{max}\right)}q_{l,j}\\ =\epsilon \frac{q_{l,j}}{2^{L-l-1}exp\left(pred_{max}\right)}. \end{array} \end{array}$$

If we choose β = min(β_*l,j*_), it always hold:


(36) $$\begin{array}{l} exp\left(q_{L-1,j}\right)-exp\left(p_{L-1,j}\right)<\epsilon \frac{q_{L-1,j}}{exp\left(pred_{max}\right)}<\epsilon . \end{array}$$

Theorem 1 is proved. By choosing a relative small value of β, the effect of the removing the incremental term *exp*() in the neuron model to the performance during inference can be controlled and minimized.

## 5. Experiments

In this section, we evaluate that the method proposed in this paper can be applied to the two problems of the TC method in RL and compare it with the general RL baseline.

Section 5.1 compares the training results of the TC and CVTC methods based on the MNIST data set and the CartPole environment. The purpose is to prove that the additional increment item β*exp*(*t*) makes all neurons always continuous and differentiable and can be removed after training without affecting network performance.

In Section 5.2, we compare the results of whether to use the coding method proposed in Section 4 and proved that the coding method is effective for RL training.

In Section 5.3, we compare our method to other baseline methods and proved that our approach could achieve the same performance as the baseline method on Benchmark tasks.

Our experiments are carried out in the CartPole-v0 and MountainCar-v0 control environment of OpenAI Gym (Barto et al., 1983), and handwritten digits data set MNIST (Mostafa, 2017; Comsa et al., 2020). All experiments run with a single process and an Nvidia RTX Quadro 5000 GPU.

### 5.1. Effectiveness of Fully Differentiable Training Method

In Section 3, we analyze the influence of increment item β*exp*(*t*) on network training and migration. In this section, we evaluate that with different β values: (1) It is effective for model training. (2) It is effective for migration. Here we don't use RL environment because the *Q*-value prediction of RL tasks needs to introduce the input coding method proposed in Section 4. To eliminate the interference, we conducted experiments based on the MNIST data set without input encoding. All grayscale images are binarized to *z*_*high*_ = 6 and *z*_*low*_ = 1 instead.

**Figure 3:**
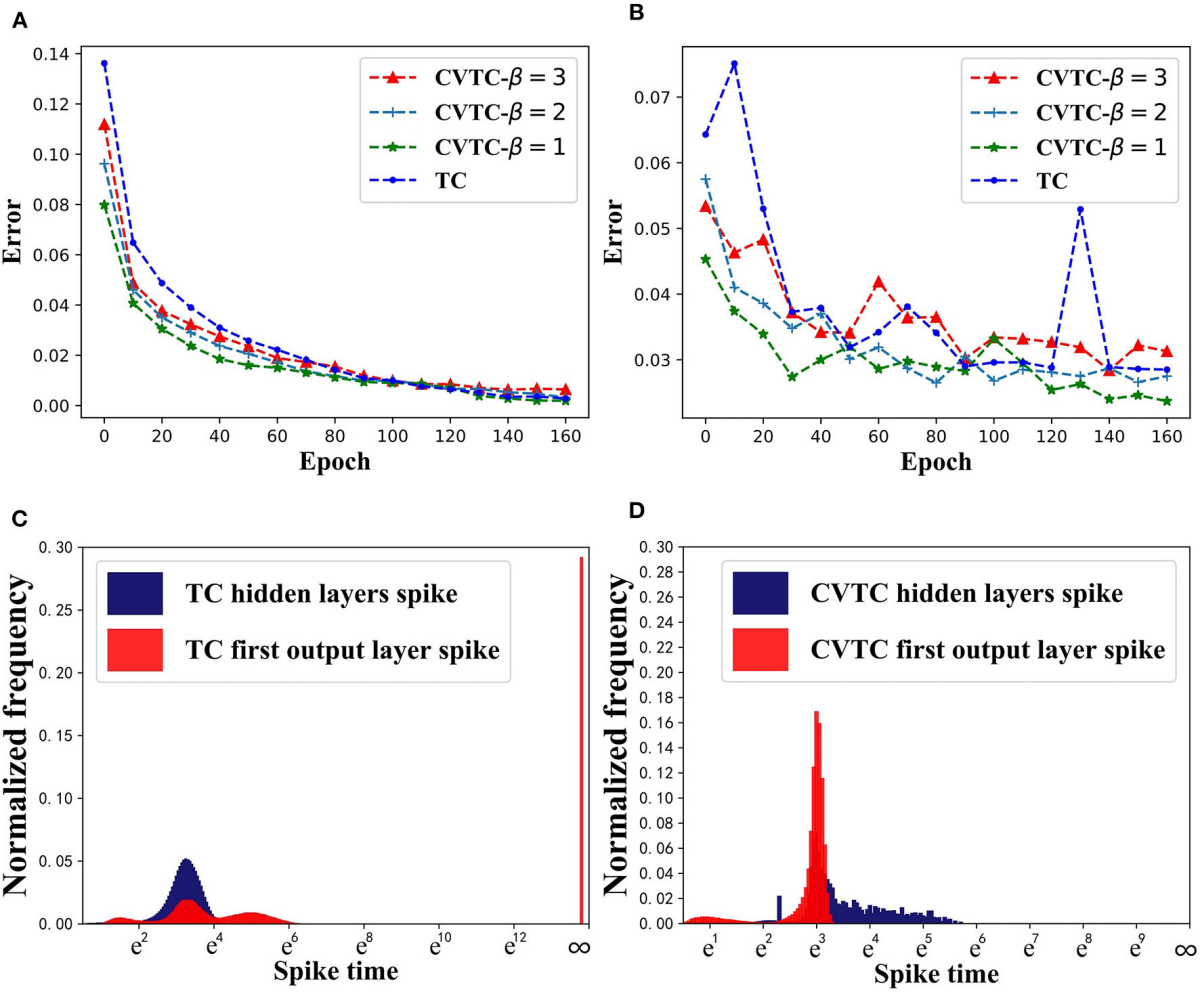
Results in MNIST task. **(A)** Training error. **(B)** Evaluation error. **(C)** Spike time distribution of TC. **(D)** Spike time distribution of CVTC.

We choose different β values, and the CVTC method was trained for 20 epochs. Figures 3A,B show the training results. Due to the increment term β*exp*(*t*), the CVTC method has a significantly faster convergence speed than the TC method. The CVTC method finally reached the same level as TC, indicating that the self-increment term introduced by the CVTC method did not affect the accuracy of algorithm training.

We show CVTC and TC methods' spike time distribution in MNIST task in Figures 3C,D. Here we use *t*_*out*_ to show the real activation time of neurons instead of *z*_*out*_. The TC method uses 1*e*6 to represent infinite *z*_*out*_, and its corresponding spike time is 19.8. As shown in Figure 3C, in a TC network, the infinity value accounts for the largest proportion in the output layer. It is far more than other values, which means the number of inactivated neurons accounts for a substantial proportion of the total. These neurons represent negative samples in classification tasks, but only positive samples and partially activated negative-sample neurons can be updated, which has a significant impact on RL tasks. As shown in Figure 3D, using our CVTC method, no illegal value appears, and the model can be updated successfully.

Then we choose different β and analyzed the effect of the inference phase (Table 3). We save the model after 50 rounds of training under different β and use the TC method to read the model parameters and verify them. The experimental results are shown in Table 3. Evaluation error refers to the result of using Equation (9), test error refers to the result of using Equation (4). It shows that as β decreases, the error in the inference stage gradually decreases. When β is less than 1*e*^−2^, the difference between the two errors approaches 0. It proves that when β is small enough, the parameters obtained by the CVTC method can be deployed to the neuromorphic chip without transformation.

**Table 3:** Comparison of errors on MNIST task.

**Beta**	**1e-1**	**1e-2**	**1e-3**
Evaluate error (%)	2.65	2.72	2.85
Test error (%)	2.67	2.72	2.85

### 5.2. Effectiveness of Input Encoding Method

In this section, we evaluate the effectiveness of the input coding method on CartPole-v0 environment. We replace the deep network with SNN in the DDQN framework, which is more stable, and proved that it would converge in finite time (Xiong et al., 2020). We use the *z*_*out*_ as *Q*-value for RL, because *z*_*out*_ has wider range than *t*_*out*_. The *Q*-value of CartPole environment has a range of [1, 200], which are included by the range of *z*_*out*_. Then the fastest responding neurons in the output layer refers to the best actions. We test the following permutations of methods: The DDQN-SNN-CVTC network using the method proposed in Sections 4 and 5.1; The DDQN-SNN-CVTC-uncoded network removes the input coding step described in Section 4; The DDQN-SNN-TC represents the original temporal coding method, which has also been introduced in Section 5.1; The DDQN-SNN-TC-encoded added the method proposed in Section 4 based on TC but did not use the network proposed in Section 3.

**Figure 4:**
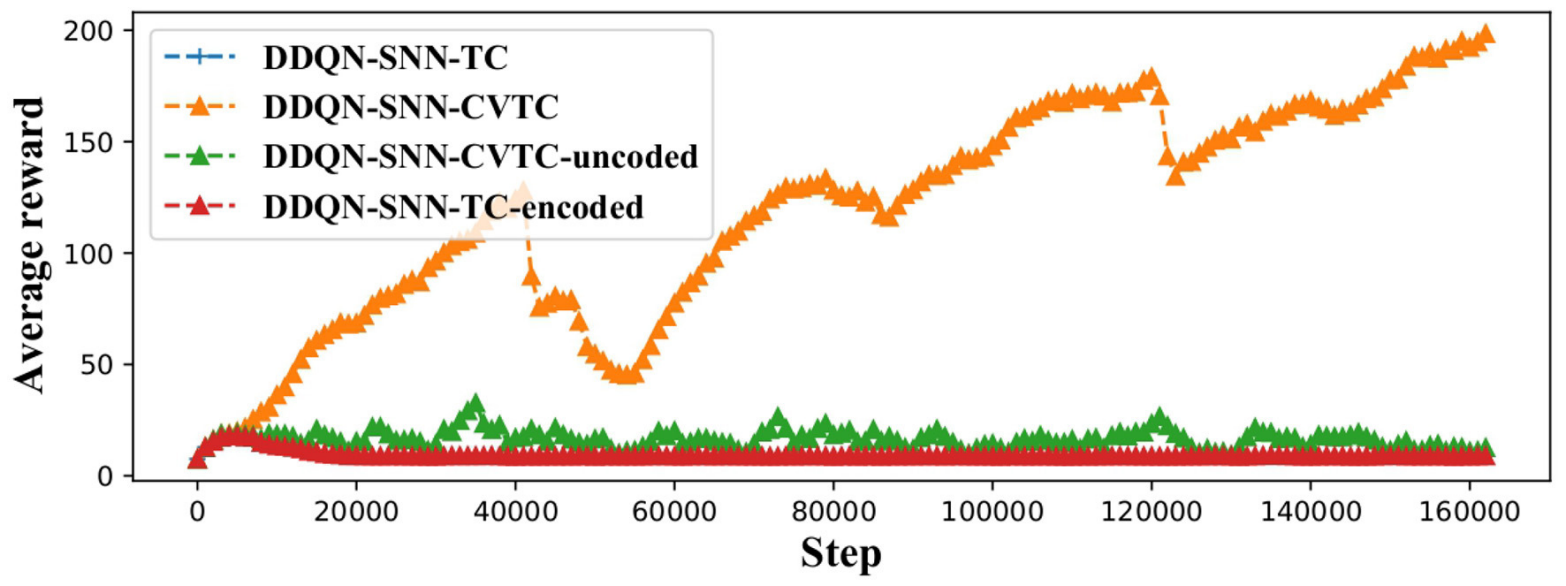
Training curves on CartPole task.

Figure 4 shows that our DDQN-SNN-CVTC out-performs all other methods on the CartPole task. Both DDQN-SNN-TC and DDQN-SNN-TC-encoded failed to learn a better-than-random policy because of the discontinuity in back-propagation. The performance of DDQN-SNN-CVTC-uncoded is better than the DDQN-SNN-TC and DDQN-SNN-TC-encoded but inferior to the DDQN-SNN-CVTC algorithm, which indicates that our input encoding method is effective for RL training.

### 5.3. Performance Evaluation

We evaluate the performance of our approach on Gym basic tasks. The MountainCar environment always returns −1 as the reward, so we need to make the reward positive to ensure the *Q*-value always greater than 1. We compare our method with two commonly used RL algorithms: DDQN for value-based RL method and proximal policy optimization(PPO) (Schulman et al., 2017) for policy-based RL method. We run all types of experiments 10 times and averaged their best rewards.

As shown in Table 4, the strategy-based PPO is obviously stronger than other methods, which may be due to the optimization method of PPO and the appropriate entropy coefficient. The DDQN-SNN-CVTC method achieved high scores, but DDQN-SNN-TC did not have any positive performance on both tasks, indicating that our method can effectively train reinforcement learning tasks. The DDQN-SNN-CVTC method is based on the same architecture as the DDQN method, but the effect is slightly inferior to the DDQN algorithm, which implies that the SNN training method still has a slight loss compared to the ANN. But in general, we have achieved an effective means of giving the advantages of the SNN method to RL.

**Table 4:** Comparison of performance on Gym basic tasks.

**Environment**	**CartPole**	**MountainCar**
DDQN	195.95 ± 0.59	−106.4 ± 1.05
PPO	198.57 ± 0.42	−96.20 ± 0.46
DDQN-SNN-CVTC (ours)	180.19 ± 2.73	−108.15 ± 2.1
DDQN-SNN-TC	17.89 ± 0.3	−199 ± 0

Currently, there are still some special challenges to train SNNs for Policy-based reinforcement learning tasks with the TC method due to the existence of two regression networks in the policy gradient algorithm: *Q*-value network and policy network. Although we can make the *Q*-value lower bound by setting the reward, the output of the policy network is unbounded, which does not match the output threshold of the SNN (0, ∞). Therefore, the next step is to provide a method for limiting the policy network to a certain threshold, so that the policy-based reinforcement learning algorithm can be used as the baseline for improvement, and the proposed method will also show better performance.

## 6. Conclusion

This paper presents the CVTC method to train asynchronous SNNs. We introduce a constantly increasing variable for each spiking neuron to ensure that it is differentiable anytime during training. This variable can be removed after training without performance degradation. Then we propose a novel temporal coding method to encode input signals with normal distribution using a group of input coding neurons. It solves the problem of losing information of later arrived spikes. Moreover, we theoretically prove that the encoded input information can be easily restored from the encoded spike times. We show that using our CVTC method, SNNs can be trained for RL tasks and achieve a comparable performance of the state-of-the-art ANN in the DDQN framework. Code can be found at: https://github.com/Dongchenl/CVTC.

## Data Availability Statement

The original contributions presented in the study are included in the article/supplementary material, further inquiries can be directed to the corresponding author/s.

## Author Contributions

GW designed the reinforment learning architecture. JW analyzed the experimental data. SL conducted the experiments. DL designed the temporal coding training algorithm. All authors contributed to the article and approved the submitted version.

## Funding

This work was supported in part by the National Natural Science Foundation of China under Grant 62002369.

## Conflict of Interest

The authors declare that the research was conducted in the absence of any commercial or financial relationships that could be construed as a potential conflict of interest.

## Publisher's Note

All claims expressed in this article are solely those of the authors and do not necessarily represent those of their affiliated organizations, or those of the publisher, the editors and the reviewers. Any product that may be evaluated in this article, or claim that may be made by its manufacturer, is not guaranteed or endorsed by the publisher.
